# Downregulation of PIK3CA via antibody-esiRNA-complexes suppresses human xenograft tumor growth

**DOI:** 10.1371/journal.pone.0200163

**Published:** 2018-07-12

**Authors:** Nicole Bäumer, Jan Rehkämper, Neele Appel, Lisa Terheyden, Wolfgang Hartmann, Eva Wardelmann, Frank Buchholz, Carsten Müller-Tidow, Wolfgang E. Berdel, Sebastian Bäumer

**Affiliations:** 1 Department of Medicine A, Hematology/Oncology, University of Muenster, Muenster, Germany; 2 Gerhard-Domagk Institute for Pathology, University of Muenster, Muenster, Germany; 3 Universitäts KrebsCentrum (UCC), Medical Systems Biology, Medical Faculty, Technische Universität Dresden, Dresden, Germany; 4 German Cancer Research Center (DKFZ), Heidelberg and German Cancer Consortium (DKTK) Partner Site, Dresden, Germany; 5 Max Planck Institute of Molecular Cell Biology and Genetics, Dresden, Germany; 6 National Center for Tumor Diseases (NCT), University Hospital Carl Gustav Carus, Technische Universität Dresden, Dresden, Germany; 7 Department of Medicine V, University of Heidelberg, Heidelberg, Germany; University of Navarra, SPAIN

## Abstract

Precision cancer therapy requires on the one hand detailed knowledge about a tumor’s driver oncogenes and on the other hand an effective targeted therapy that specifically inhibits these oncogenes. While the determination of genomic landscape of a tumor has reached a very precise level, the respective therapy options are scarce. The application of small inhibitory (si) RNAs is a promising field of investigation. Here, we present the effective *in vivo*-treatment of colorectal cancer (CRC) xenograft tumors with antibody-complexed, endoribonuclease-prepared small inhibitory (esi)RNAs. We chose heterogeneous endoribonuclease-prepared siRNA pools (esiRNAs) against the frequently mutated genes PIK3CA and KRAS and coupled them to the anti-EGFR antibody cetuximab, which was internalized specifically into the tumor cells. esiRNA pools have been shown to exhibit superior specificity in target gene knockdown compared to classic siRNAs. We identified a significant decrease in tumor growth upon this treatment due to decreased tumor cell proliferation. The *ex vivo*-analysis of the xenograft CRC tumors revealed the expected downregulation of the intended direct targets PIK3CA and KRAS on protein level. Moreover, known downstream targets for EGFR signaling such as p-ERK, p-AKT, and c-MYC were decreased as well. We therefore propose the use of antibody-esiRNA complexes as a novel experimental treatment option against key components of the EGFR signaling cascade.

## Introduction

Tumor cell growth and survival often depend on constitutively active signaling pathways such as epidermal growth factor receptor (EGFR) signaling. Therefore, the inhibition of such an activated pathway is an attractive target for cancer therapy [1]. The chimeric IgG1 monoclonal antibody cetuximab inhibits the EGF receptor directly by receptor blocking and leads to a significantly prolonged survival of colorectal patients [2,3]. However, signaling pathway activation can be acquired by activating mutations of key components of these pathways downstream of the respective receptor, which then counteracts an EGFR targeted therapy [4,5]. In colorectal cancer (CRC), mutations of EGFR downstream targets occur especially in the GTPase KRAS in up to 45% and in the phosphatidylinositol-4,5-bisphosphate 3-kinase, catalytic subunit alpha (PIK3CA) in up to 30% of patients [4,6–8]. These factors transfer signals from activated EGF receptor intracellularly mainly via two different branches: the RAS-RAF-MAPK and PI3K-AKT-mTOR pathways [9]. Third, a contribution of Wnt signaling downstream EGFR is discussed [10].

PIK3CA mutations are the most common in cancer and PIK3CA is the most frequently mutated kinase in the human genome [11,12]. The phosphatidylinositol 3-kinase (PI3K) is composed of an 85 kDa regulatory subunit and a 110 kDa catalytic subunit PIK3CA, which uses ATP to phosphorylate phosphatidylinositols. The three hotspot mutation positions (GLU542 and GLU545 in exon 9, and HIS1047 in exon 20) have been reported for several cancers. Its oncogenic function is underlined by the fact that the kinase domain mutated PIK3CA-H1047R induces mammary gland tumors in mouse knock-in models [13,14] and colon carcinoma in a transgenic mouse model [15] and the helicase mutated PIK3CA E545K mammary tumors [16].

The obvious implication of activated PIK3CA in cancer lead to numerous studies concerning the impact of PIK3CA mutations in prognosis and therapy outcome. Mutations of PIK3CA are associated with clinical resistance to EGFR-targeted therapies [17], an example is PIK3CA-H1047R [18]. The role of PIK3CA mutations for overall survival and progression-free survival in CRC are stated as neutral [19] or associated with poor prognosis [20].

However, due to the high abundance in different cancer entities, oncogenic mutated PIK3CA is an attractive target for cancer therapy. Many attempts are ongoing to develop functional and specific small molecule inhibitors against activated kinases and especially PIK3CA [12,21,22]. The only FDA-approved PI3K inhibitors to date are the small molecule PI3K-delta inhibitors idelalisib and copanlisib, [12,22–26]. Despite many attempts to establish a functional inhibitor of PIK3CA, ongoing studies imply that a monotherapy might not be effective enough and that the selection of patients might be very important to combine different therapeutic agents according to the patients’ target requirements [12,21,27–29].

Generally, application of small molecule inhibitors might lead to the development of resistant cancer cell clones [30]. The use of the RNAi technology to downregulate key oncogenes could represent a more specific and effective treatment option [31,32]. *In vivo* RNAi requires the transport of siRNA into cancer cells without degradation in the blood flow and ideally a transfer into the intracellular compartment via a cancer cell specific uptake mechanism. Different strategies to deal with these requirements were discussed in our recent review [33]. To achieve this, we developed a system to couple esiRNA to the cancer cell-specific anti-EGFR-antibody cetuximab [34,35] which delivers esiRNA to the intended cancer cells, binds to the EGFR receptor and gets internalized in a receptor-dependent fashion. Here, we will describe the results of a study aiming for the simultaneous siRNA interference of MAPK and PI3K signaling pathways both downstream the EGFR receptor by a cetuximab-esiRNA carrier system in colorectal cancer *in vitro* and *in vivo*.

## Materials and methods

### Coupling of anti-EGFR monoclonal antibody to protaminesulfate

Protamine sulfate (4 mM) was amino-terminally coupled to the bifunctional crosslinker Sulfo-SMCC (sulfosuccinimidyl 4-(N-maleimidomethyl)cyclohexane-1-carboxylate) (Pierce No. 22622, Rockford, IL, USA) in a 1:4.5 molar ratio in amino-free PBS buffer. The pH was adjusted to pH 7 with 0.1 M carbonate buffer (pH 8.3). The mixture was left to react for 1h at room temperature (RT), then coupled to cysteine residues of anti-EGFR monoclonal antibody cetuximab (31 μM stock; Erbitux^™^, Merck-Serono, Darmstadt, Germany) in a 32:1 molar ratio at 4°C overnight. Non-reacted educts and protamine doublets were separated from the high molecular weight cetuximab-protamine product by gel filtration chromatography in Zeba spin desalting columns (Pierce No. 89891). The cetuximab-protamine adduct was stored at 4°C and was stable for several weeks.

### siRNAs and esiRNAs

For the estimation of siRNA coupling, stability and internalization efficiency, cetuximab-protamine was coupled to Allstars negative control siRNA-Alexa 488 (“scrambled”, cat. no. 1027284, Qiagen, Hilden, Germany) or Allstars negative control siRNA-Alexa 555 (cat. no. 1027286, Qiagen, Hilden, Germany). Treatment experiments were done using esiRNA duplexes against KRAS (KRAS-Mission esiRNA, EHU114431), PIK3CA esiRNA (EHU039391) and as a control anti-GFP esiRNA (EHU-GFP, all Eupheria Biotech, Dresden).

### Coupling of (e)siRNA to cetuximab-protamine

siRNA duplexes were bound to cetuximab-protamine in a 4 to 10-fold molar excess at RT for 1–2 hours. This complex was prepared freshly before use.

### Cell culture

HT29 were cultivated in IMDM, SW480 and DLD1 cells in RPMI medium, all supplemented with 10% fetal calf serum (FCS), 1% streptomycin and penicillin and 1% glutamine. Cell lines harbor mutations as depicted in S1 Table. Cell lines were obtained from ATCC (Manassas, VA) or DSMZ (Braunschweig, Germany) and routinely tested for identity by morphological analysis, cell surface receptor FACS analysis and were only used low-passage.

### Fluorescent microscopy

DLD1, SW480 and HT29 cells were seeded at 2x10^4^ cells/cm^2^, cultivated on 4 well-chamber slides (Sigma C7057, Taufkirchen, Germany) over-night and treated with anti-EGFR monoclonal antibody cetuximab-protamine or cetuximab alone incubated with Alexa Fluor 488-labeled Allstars control siRNA (Qiagen 1027284, Hilden, Germany) and Allstars negative control siRNA-Alexa 555 (Qiagen 1027286, Hilden, Germany) at 1:10 molar ratio each over night at 37 °C and 5% CO_2_. Subsequently, cells were washed with phosphate buffered saline (PBS), fixed with ice-cold paraformaldehyde (PFA) 4%, stained with Hoechst, mounted with Dako fluorescent mounting medium and photographed on a Zeiss Axioskop.

#### Western blots

5x10^5^ cells of each cell line were seeded and cultivated overnight, treated with cetuximab-protamine (60 nM) coupled to the indicated esiRNAs at 1:10 molar ratio once a day for 72 h, harvested, lysed in RIPA buffer and cleared by centrifugation. Xenograft tumors were homogenized as 10% w/v in RIPA buffer using an ultraturrax, cleared by ultrasonification and centrifugation. Western blot analysis was performed using standard protocols with the following antibodies: anti-KRAS (ab55391, ABCAM, Cambridge, UK), PI3Kinase- p110α (4249), anti-ERK1/2 (4696), anti-phospho-ERK1/2 (4370), p-AKT-S473 (4058), anti-AKT, anti c-MYC (9402, all Cell Signaling Technology, Danvers, MA, USA), and anti β-Actin antibody (Clone AC-15, Sigma Aldrich). Densitometric analysis of gel-electrophoretic bands was carried out using the NIH Image J package (http://rsb.info.nih.gov/ij/).

### Clonogenic growth in soft agar

In brief, 4,000 trysinized cells in 120 μl per sample were incubated with cetuximab-protamine coupled to the indicated siRNAs at 60 nM end concentration for one hour at RT, resuspended in 168 μl of 3% agar (Difco Agar Noble) and 432 μl medium plus 10% FCS, 1% streptomycin and penicillin, 1% glutamine, and cultivated in triplicate for colony formation in 96-well format (180 μl/well). A second treatment with 60 nM end concentrations was performed after seven days of culture. After two weeks, the assays were stained with 1 mg/ml INT (Iodonitrotetrazolium chloride) solution and incubated over night at 37°C. The next day the assays were counted for colony numbers.

### Mouse xenograft tumor model

All animal experiments in this study were carried out in accordance with the recommendations of the Institutional Animal Care and Use Committee ‘‘Landesamt fuer Natur, Umwelt und Verbraucherschutz NRW” (LANUV). This study was performed with permission of the Institutional Animal Care and Use Committee and of the local veterinary administration of Muenster (Permit Number 84–02.04.2014.A285).

Mice were kept in individually ventilated (IVC-) Typ II cages (Tecniplast GmbH, Germany) in groups of five mice, in a 12-hour light/dark cycle, with room temperature at 22±2 °C and a relative air humidity of 45–65%. All mice were allowed free access to water and a maintenance sterile diet. All reasonable efforts were made to ameliorate suffering, including isolation of affected mice. Mice were monitored daily for signs of pain or distress. Moribund mice were humanely sacrificed as described below. Study design and biometric planning of each experiment was performed in accordance with a biostatistician. Mice were sacrificed for sample preparation by deep anesthesia via CO_2_ inhalation followed by cervical dislocation. For each experiment, the single animal was an experimental unit.

Female CD1 nude mice (Charles River, Wilmington, MA, USA) were transplanted subcutaneously in the flank region with 1x10^7^ HT29, DLD1, or SW480, respectively, and kept until tumors reached sizes of approximately 200 mm^3^. Mice were randomized into groups of 6 and treated with cetuximab-protamine coupled to the respective (e)siRNA, or cetuximab-protamine at 4 mg/kg twice a week intraperitoneally. Tumor growth was followed with caliper measurements and tolerance for the animal was assessed by measuring body weight and scoring overall appearance. Tumor volumes were calculated by the formula length × width^2^ × 0.52 [36]. At the end of the experiment, animals were euthanized, tumors were isolated, and tumor weight was determined.

### Immunostainings of xenograft tumors

For immunostaining, the tumors were embedded in paraffin using standard methods. For Ki67, KRAS and PIK3CA staining, dehydrated sections were blocked with 3% BSA, and stained with anti Ki67 antibody (clone MIB-1, DakoCytomation, Glostrup, Denmark), anti KRAS antibody (clone 9.13, ThermoFisher Scientific, Schwerte, Germany), or anti PIK3CA antibody (PI3Kinase-p110α (4249, Cell Signaling Technology, Danvers, MA, USA),) processed with mouse anti rabbit-peroxidase, washed and developed according to standard methods. Counterstain was performed using hematoxylin using standard methods.

For c-MYC and KRAS immunofluorescence, dehydrated sections were boiled for 3 min in 10 mM citric acid, 0.05% Tween 20, pH 6.0, washed twice in PBS and blocked in 1.5% normal goat serum, 0.1% Tween 20 in PBS. Sections were incubated with c-MYC (clone Y69, Abcam, Cambridge, UK; 1:500) and KRAS (ab55391, Abcam, Cambridge, UK; 1:100) antibody diluted in blocking solution overnight at 4°C, washed three times in PBS/0.1% Tween 20 for 5 min, incubated with secondary antibodies (goat anti-rabbit-Cy3 and donkey anti-mouse-Alexa 488, 1:500 each) diluted in blocking solution for 1–2 h at RT, counterstained with Hoechst 33342 and mounted using Dako mounting medium.

### Statistical analysis

All data are presented as means ± standard deviation, if not indicated otherwise. The mean values of two groups were compared by 2-tailed Student's t test.

## Results

In our first study, we analyzed the effect of KRAS downregulation [35] which lead to significantly decreased tumor growth in KRAS mutated colorectal cancer xenograft tumors. However, tumors still grew upon treatment, and as expected, BRAF-mutated HT29-tumors did not respond to KRAS inhibition because BRAF acts downstream of KRAS in the MAPK pathway. Here, we present our results on the application of PIK3CA esiRNA with and without combined knockdown of KRAS.

### CRC cell lines are accessible for a combination treatment with two siRNAs

**Fig 1:**
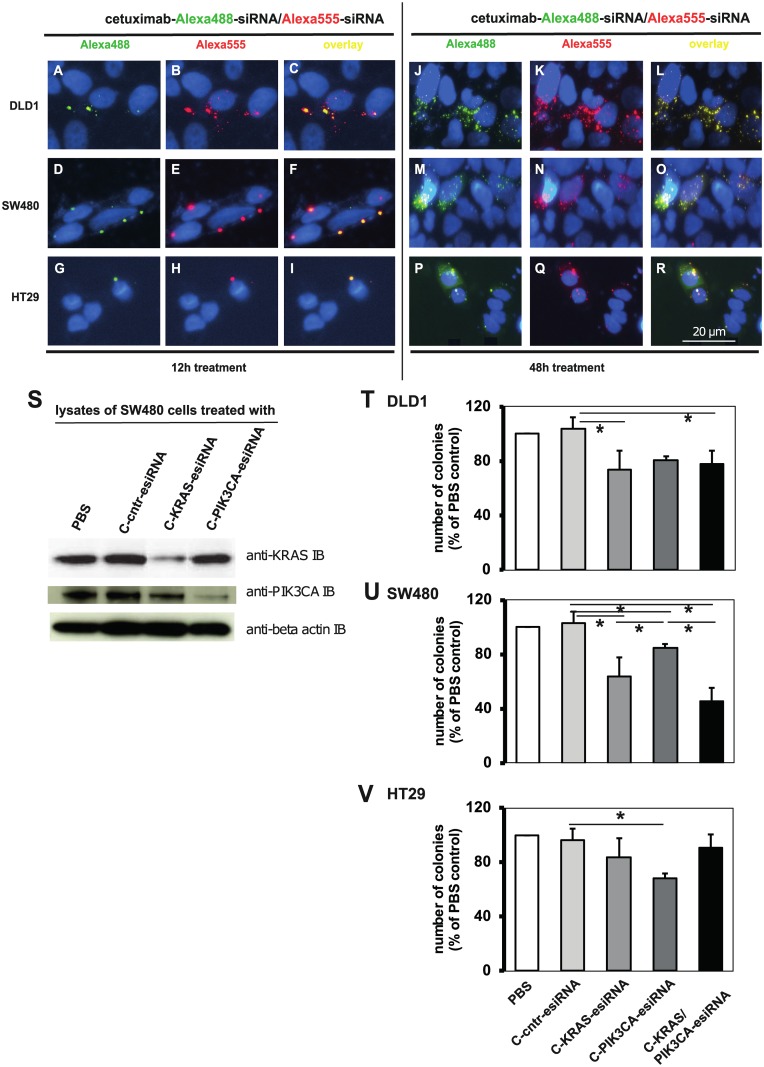
Cetuximab-sulfo-SMCC-protamine ability to transport two independent siRNAs. **A-I**. Alexa 488 and Alexa 555 control-siRNA were mixed, coupled to cetuximab-sulfo-SMCC-protamine and incubated with the indicated cell lines at 60 nM end concentration overnight (Fig 1 A to I) or 48 h (J to R). Cells were washed, fixed, stained with Hoechst and mounted. Fluorescent micrographs showed vesicular enrichment of both Alexa488 and -555 signals within the same vesicles. **S**. SW480 cells were treated with cetuximab (“C”)-sulfo-SMCC-protamine coupled to control- (“C-cntr-esiRNA”), KRAS-specific or PIK3CA-specific esiRNAs at 60 nM end concentration for 3 consecutive days. Cells were washed, harvested and lysates were subjected to Western blot against KRAS, PIK3CA and actin as loading control. C-KRAS-esiRNA treatment reduced KRAS protein expression, whereas C-PIK3CA-esiRNA treatment reduced PIK3CA protein expression.**T—V**. Soft agar colony forming assays of CRC cell lines DLD1 (**T**) and SW480 (**U**) treated with cetuximab-coupled control esiRNA ("C-scrm" = C-cntr-esiRNA), anti-KRAS-, anti-PIK3CA- or both esiRNAs. Results showed significant reduction of colony growth with C-KRAS-esiRNA and C-PIK3CA-esiRNA, but only SW480 showed additive effect with both esiRNAs. HT29 (BRAF-mutated, PIK3CA-mutated) cells (**V**) were affected by C-PIK3CA-esiRNA, but not by C-KRAS-esiRNA treatment. Shown are number of colonies normalized to PBS-treated cells of three independent experiments, mean +/- SD. * p<0.05.

First, we tested the ability of the antibody-protamine complex to bind and carry two different siRNAs and internalize them into the cells in a receptor-dependent fashion. We coupled classic control siRNA that was labeled with Alexa 488 and/or Alexa 555, respectively to the anti-EGFR-antibody cetuximab via sulfo-SMCC-protamine and treated the colorectal cancer (CRC) cell lines DLD1, SW480 and HT29 with these antibody-siRNA complexes *in vitro*. All cell lines tested showed fluorescent vesicular structures within their cytoplasm positive for Alexa 488- (green, Fig 1A, 1D and 1G) and Alexa 555-positive after 12 h treatment (red, Fig 1B, 1E and 1H; overlays in Fig 1C, 1F and 1I). However, as most experimental assays were characterized by a much longer treatment period, we also performed this targeting for a 48 h timeframe. Here, a considerable higher degree of siRNA targeting was visible (Fig 1J–1R) in all cell lines. We conclude that the anti-EGFR-antibody cetuximab is able to transport mixtures of different siRNAs simultaneously and in a trackable amount into cells.

Since we chose PIK3CA as a target for our study, we exemplarily treated SW480 cells with cetuximab-protamine complexed with anti-PIK3CA-esiRNA and identified a knockdown of PIK3CA protein expression in esiRNA treatment (“C-PIK3CA-esiRNA”), Fig 1S, middle row). In turn, KRAS expression was downregulated by cetuximab-KRAS-esiRNA treatment (“C-KRAS-esiRNA”, top row). With these *in vitro* results, we had the rationale to test the effect of combined siRNA transported by cetuximab on colony formation ability in soft agar assays.

### Inhibition of KRAS and PIK3CA in complex with cetuximab leads to decreased colony formation

Colony formation in soft agar is a well-established landmark for the tumorigenic growth of cell lines [35]. In our previous study, we observed that even a moderate reduction of colony growth modeled a strong inhibition of xenograft tumor growth, which indicates that this assay is indeed relevant as an indicator for *in vivo* tumorigenic growth in our treatment setting.

As expected, cetuximab-protamine coupled to KRAS-esiRNA alone reduced clonogenic growth only in KRAS-mutant DLD1 and SW480 cells (Fig 1T and 1U). Colony growth of the KRAS-wild type cell line HT29 was independent of cetuximab-KRAS-esiRNA treatment (Fig 1V). Remarkably, treatment with cetuximab-PIK3CA-esiRNA complexes lead to significantly decreased colony growth in PIK3CA-mutant HT29 as well as in PIK3CA-wild type SW480 cells (Fig 1U and 1V). Colony formation of SW480 and PIK3CA-mutant DLD1 cells was also sensitive to a combination of cetuximab-KRAS-esiRNA and PIK3CA-esiRNA double-complex (Fig 1T and 1V). Interestingly, HT29 colony growth was less inhibited by the esiRNA combination than by C-PIK3CA-esiRNA alone Fig 1V), indicating that in the combination the carrier capacity for active single components may be critical. These results indicate that we succeeded in treating cells with a PIK3CA-inhibiting antibody-esiRNA complex and that the expression of PIK3CA, independent of its mutation status, is an important factor for tumorigenic *in vitro* growth for certain cell types. This prompted us to investigate the efficiency of cetuximab-PIK3CA-esiRNA complex treatments in xenograft mouse models *in vivo*.

### KRAS and PIK3CA-esiRNA-anti-EGFR complexes are efficient in human CRC xenografts

**Fig 2:**
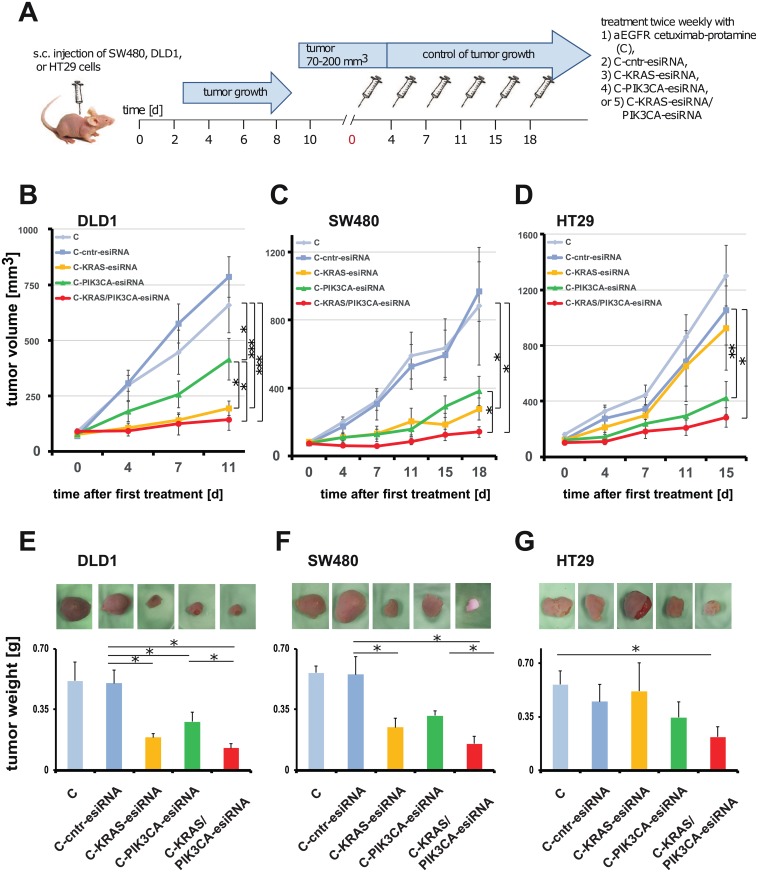
CRC xenograft growth control by cetuximab-sulfo-SMCC-protamine coupled to control-, KRAS- and/or PIK3CA-esiRNA treatment. **A**. Time schedule and treatment regimen. CRC cell lines were transplanted subcutaneously to CD1 nude mice and tumor growth observed to a size as indicated. Treatment started day 0 after randomization of the animals to the different groups and was continued twice a week by i.p. applications of PBS, cetuximab-sulfo-SMCC-protamine coupled to control esiRNA (C-cntr-esiRNA) or the indicated esiRNAs directed against KRAS (C-KRAS-esiRNA) and PIK3CA (C-PIK3CA-esiRNA) or both (C-KRAS/PIK3CA-esiRNA). C-KRAS-esiRNA treatment was effective in DLD1 (**B**) and SW480 tumor (**C**) size control, but not in HT29 (BRAF-mutated, PIK3CA-mutated; **D**) as compared to controls. C-PIK3CA-esiRNA treatment was effective in SW480 and HT29, but less effective in DLD1. C-KRAS/PIK3CA-esiRNA combination treatment reduced tumor growth in the xenografts of all cell lines. Graph shows median tumor volumes normalized to day 0 +/- SEM. Detailed statistical analysis is presented in Table 1. Growth curves and tumor weight for C, C-cntr-esiRNA (C-scr in E-G) and C-KRAS-esiRNA treatment in **B-G** have been published before [35] and are reproduced here as controls with permission of the AACR to reduce the number of control animals according to the 3R rules [37]. **E-G**. *Ex vivo* photographs of dissected tumors and excised tumor weight statistics reveal significant reduction of tumor size and weight compared to controls in C-KRAS-esiRNA treated DLD1 and SW480 tumors, but not in HT29. In turn, C-PIK3CA-esiRNA reduced tumor weight significantly in all tumors along with additive effects of C-KRAS/PIK3CA-esiRNA combination treatment. Graph shows absolute tumor weight +/- SD. Significance * = p < 0.05.

Next, we analyzed the effect of cetuximab-esiRNA complexes in human CRC xenografts *in vivo*. Briefly, we injected 1x10^7^ cetuximab-resistant DLD1, SW480 or HT29 colorectal carcinoma cells, respectively, subcutaneously into the flank of CD1 nude mice. According to the “3R”-rules reviewed in [37], we performed these experiments using the same cetuximab-protamine (C), C-cntr-esiRNA, and C-KRAS-esiRNA treatment groups as published in our previous study [35] and simultaneously performed treatments with C-PIK3CA-esiRNA and C-KRAS-esiRNA/PIK3CA-esiRNA combinations (Fig 2). The mice were injected intraperitoneally twice a week with the respective antibody-complexes (see Fig 2A for schematic overview).

In all three human xenograft models, we observed a strong inhibitory effect of C-PIK3CA-esiRNA on tumor growth (for details also see Table 1). In HT29 tumors, the C-PIK3CA-esiRNA (green) treatment was more efficient than the C-KRAS-esiRNA treatment (yellow, Fig D; Table 1). However, a combination of C-KRAS/PIK3CA-esiRNA lead to significantly smaller tumor size in HT29 compared to control groups (Fig 2D; Table 1). Taken together, in all three models, the most efficient growth inhibition of xenografts was achieved with a combination C-KRAS/PIK3CA-esiRNA (red, Fig 2B–2D; Table 1), indicating that the combined inhibition of two branches of the EGFR-signaling pathway is very effective in slowing down tumor growth.

**Table 1:** Xenograft tumor size as determined by caliper measurement during treatment.

**DLD1**						
**Mean (mm**^**3**^**) ± SEM**						
	**Day 0**	**Day 4**	**Day 7**	**Day 11**		
Cetuximab-sulfo-SMCC-protamine (C)	90.1±11.1	297.6±68.9	446.3±99.9	659.3±124.8		
C-cntr-esiRNA	69.2±5.7	307.6±35.8	575.0±87.6	785.3±91.6		
C-KRAS-esiRNA	77.6±15.3	106.1±22.4	141.8±25.9	193.9±31.8		
C-PIK3CA-esiRNA	78.6±15.0	179.8±45.3	256.2±60.4	415.0±92.9		
C-KRAS/PIK3CA-esiRNA	90.5±11.5	93.4±24.0	124.4±50.4	144.1±50.1		
**t-test**						
t-test C-cntr-esiRNA vs C-KRAS-esiRNA	n.s.	0.0007	0.0008	0.0001		
t-test C-cntr-esiRNA vs C-PIK3CA-esiRNA	n.s.	n.s.	0.0132	0.0173		
t-test C-KRAS-esiRNA vs C-KRAS/PIK3CA-esiRNA	n.s.	n.s.	n.s.	n.s.		
t-test C-PIK3CA-esiRNA vs C-KRAS/PIK3CA-esiRNA	n.s.	n.s.	n.s.	0.0275		
t-test C-cntr-esiRNA vs C-KRAS/PIK3CA-esiRNA	n.s.	0.0005	0.0012	0.0001		
t-test C-KRAS-esiRNA vs C-PIK3CA-esiRNA	n.s.	n.s.	n.s.	0.0473		
**SW480**						
**Mean (mm**^**3**^**) ± SEM**						
	**Day 0**	**Day 4**	**Day 7**	**Day 11**	**Day 15**	**Day 18**
Cetuximab-sulfo-SMCC-protamine (C)	83.7±11.6	200.7±43.0	324.9±92.5	590.4±131.1	634.3±146.8	881.4±174.8
C-cntr-esiRNA	73.0±12.8	173.6±29.0	304.6±55.7	525.7±136.9	594.1±171.7	966.7±345.9
C-KRAS-esiRNA	81.7±12.4	109.3±26.8	130.3±45.3	202.0±78.1	185.6±64.8	276.2±64.8
C-PIK3CA-esiRNA	77.0±6.2	109.1±16.5	129.1±27.5	157.8±40.8	291.9±63.2	382.5±85.7
C-KRAS/PIK3CA-esiRNA	72.1±2.6	60.5±10.5	58.0±10.3	85.8±19.6	124.7±32.4	141.4±32.5
**t-test**						
t-test C-cntr-esiRNA vs C-KRAS-esiRNA	n.s.	n.s.	0.0501	0.0493	0.0494	0.0472
t-test C-cntr-esiRNA vs C-PIK3CA-esiRNA	n.s.	n.s.	0.0270	0.0400	n.s.	n.s.
t-test C-KRAS-esiRNA vs C-KRAS/PIK3CA-esiRNA	n.s.	n.s.	n.s.	n.s.	n.s.	n.s.
t-test C-PIK3CA-esiRNA vs C-KRAS/PIK3CA-esiRNA	n.s.	0.0461	n.s.	n.s.	0.0397	0.0247
t-test C-cntr-esiRNA vs C-KRAS/PIK3CA-esiRNA	n.s.	0.0072	0.0025	0.0154	0.0224	0.0383
t-test C-KRAS-esiRNA vs C-PIK3CA-esiRNA	n.s.	n.s.	n.s.	n.s.	n.s.	n.s.
**HT29**						
**Mean (mm**^**3**^**) ± SEM**						
	**Day 0**	**Day 4**	**Day 7**	**Day 11**	**Day 15**	
Cetuximab-sulfo-SMCC-protamine (C)	159.8±13.3	326.1±46.7	444.1±72.1	862.8±158.8	1302.8±218.7	
C-cntr-esiRNA	122.9±15.7	276.1±79.3	346.2±79.3	684.2±176.2	1053.6±251.4	
C-KRAS-esiRNA	122.1±17.8	211.1±49.3	297.8±77.8	653.1±254.0	925.4±301.8	
C-PIK3CA-esiRNA	119.5±19.2	143.5±31.7	240.1±73.0	295.8±80.7	423.5±116.9	
C-KRAS/PIK3CA-esiRNA	100.6±16.7	109.9±23.4	184.4±53.7	209.2±56.1	282.5±69.6	
**t-test**						
t-test C-cntr-esiRNA vs C-KRAS-esiRNA	n.s.	n.s.	n.s.	n.s.	n.s.	
t-test C-cntr-esiRNA vs C-PIK3CA-esiRNA	n.s.	n.s.	n.s.	n.s.	n.s.	
t-test C vs C-PIK3CA-esiRNA	n.s.	0.0141	n.s.	0.0154	0.0087	
t-test C-KRAS/PIK3CA-esiRNA vs C-KRAS-esiRNA	n.s.	n.s.	n.s.	n.s.	n.s.	
t-test C-KRAS/PIK3CA-esiRNA vs C-PIK3CA-esiRNA	n.s.	n.s.	n.s.	n.s.	n.s.	
t-test C-KRAS/PIK3CA-esiRNA vs C-cntr-esiRNA	n.s.	n.s.	n.s.	0.0403	0.0220	
t-test C-PIK3CA-esiRNA vs C-KRAS-esiRNA	n.s.	n.s.	n.s.	n.s.	n.s.	

Shown here are tumor volumes in mm3 and statistical significance between the different treatment groups (2-sided t-test).

Upon termination of the experiment, when the first animal in the experiment reached an intolerable tumor size according to animal welfare guidelines, we sacrificed the mice and prepared the tumors for further in depth-analysis. First, we determined excised tumor weight, which correlated well with the measured sizes at the last treatment time point (Fig 2E–2G, C-scr = C-cntr-esiRNA)). Tumors were then subjected to siRNA target gene response analysis by immunostainings and Western blots.

### Tumor cell proliferation in xenograft tumors decreases upon PIK3CA inhibition

**Fig 3:**
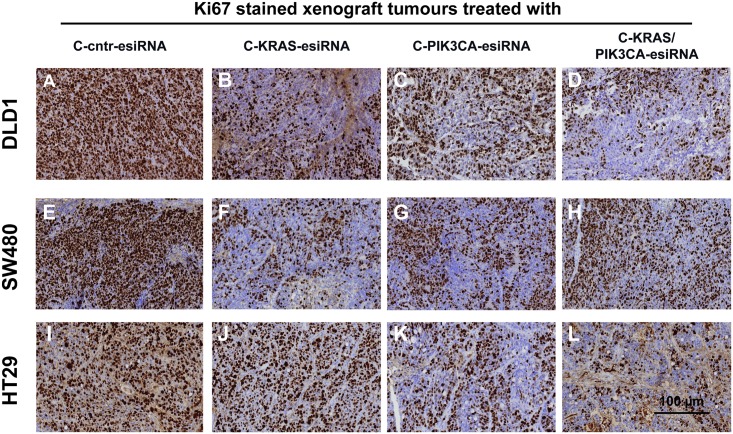
Treatment of CRC xenografts with cetuximab-protamine siRNA controls tumor cell proliferation. Immunohistochemical (IHC) analysis of Ki67 proliferation marker in treated tumor sections. Tumor samples from the experiments described in Fig 2 were embedded in paraffin, sectioned by microtome, rehydrated and subjected to IHC for Ki67 marker. Sections are presented at 20x magnification. All tumors treated with control esiRNA coupled to cetuximab (**A, E, I**: C-cntr-esiRNA) showed Ki67 nuclear signals, whereas C-KRAS-esiRNA treatment reduced Ki67 staining markedly in DLD1 (**B**) and SW480 (**F**), but not in HT29 (BRAF-mutated, PIK3CA-mutated; **J**). C-PIK3CA-esiRNA treatment reduced Ki67 staining in all tumor groups (**C, G, K**) with some further reduction by C-KRAS/PIK3CA-esiRNA combination treatment (**D, H**). In HT29 samples KI67 staining was further reduced by the combination (**L**) as compared to C-PIK3CA-esiRNA (**K**).

First, we addressed the question if the inhibited tumor cell proliferation observed after KRAS-esiRNA-treatment of human CRC xenografts ([35] and Fig 2) is also the cause for decreased tumor growth in cetuximab-antibody-coupled PIK3CA-esiRNA (C-PIK3CA-esiRNA) treatment. Tumors of xenografted DLD1, SW480 and HT29 cell lines were subjected to immunohistochemical staining to detect the standard proliferation marker Ki67 (Fig 3). As expected, DLD1 and SW480 tumors treated with cetuximab-KRAS-esiRNA (C-KRAS-esiRNA) showed less Ki67 positive cells (Fig 3B and 3F) than sections of the control group (Fig 3A and 3E), while HT29 tumors treated with control (Fig 3I) or KRAS-esiRNA-antibody complexes (Fig 3J) depicted comparable numbers of Ki67 positive cells. However, Ki67 staining was clearly decreased all three tumor types upon treatment with C-PIK3CA-esiRNA treatment (Fig 3C, 3G and 3K) as well as in the combination group which received C-KRAS/PIK3CA-esiRNA complexes (Fig 3D, 3H and 3L). Therefore, decreased tumor growth as observed *in vivo* (Fig 2) can be partly explained by inhibition of tumor cell proliferation as indicated by the marker Ki67.

### Knockdown of target proteins KRAS and PIK3CA in tumors upon cetuximab-esiRNA-complex treatment

**Fig 4:**
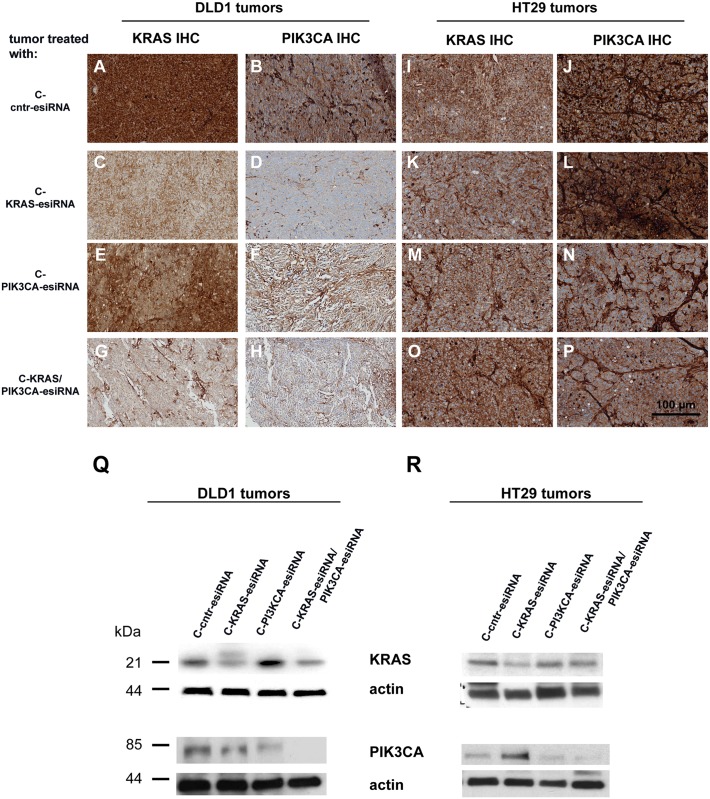
Treatment of CRC xenografts with cetuximab-protamine-siRNA markedly reduces siRNA target gene expression. **A-P**. Paraffin sections from DLD1 tumors (**A-H**) and HT29 tumors (**I-P**) were prepared for immunohistochemical (IHC) analysis for siRNA targets KRAS and PIK3CA with antibody detecting KRAS (A, C, E, G and I, K, M, O) and PIK3CA (B, D, F, H and J, L, N, P) combined with suitable secondary antibodies and stained with diaminobenzidine (DAB) and hematoxylin and pictures taken at 20x magnification from regions without signs of necrosis. The application of KRAS siRNA coupled to cetuximab-sulfo-SMCC-protamine (C-KRAS-esiRNA) markedly reduced KRAS immunostaining **(C)** in DLD1 tumors, but not in HT29 (BRAF-mutated, PIK3CA-mutated; **K**) compared to control C-cntr-esiRNA **(A, I)**. The treatment with C-PIK3CA-esiRNA reduced PIK3CA immunostaining in DLD1 (**F**) as well as HT29 tumors (**N**), as compared to control C-cntr-esiRNA in both cell lines (**B, J**). Interestingly, C-KRAS-esiRNA treatment also reduced PIK3CA staining in DLD1 tumors (**D**), but not in HT29 tumors (**L**). The combination treatment of tumors with KRAS- and PIK3CA-esiRNAs (C-KRAS/PIK3CA-esiRNA) resulted in even less KRAS and PIK3CA staining than the C-KRAS-esiRNA treatment in DLD1 tumors (**G-H**) and similar stainings of both in HT29 cells(**O-P**) as the C-PIK3CA-esiRNA monotherapy (**M-N**). **Q-S**. Western blots indicating siRNA target gene induced protein synthesis control in xenograft tumor tissue of cetuximab-protamine-esiRNA treated mice. Tumor tissue was processed for western blot as described, applied to SDS-PAGE, blotted and exposed for immunodetection by antibodies raised against KRAS, PIK3CA and actin as loading control. Application of cetuximab-protamine coupled to KRAS-esiRNA (C-KRAS-esiRNA) reduced KRAS protein levels in DLD1 (**Q**, top row), HT29 (**R**, top row) and SW480 (**S, top row**) tumor xenografts as compared to controls (actin row). In addition, C-KRAS-esiRNA treatment showed certain crosstalk to PI3K pathway signaling (third row from above) as indicated by reduced PIK3CA expression in DLD1 (**Q**), enhanced PIK3CA expression in HT29 (**R**) tumors and indifferent PIK3CA expression effect in SW480 (**S**) as compared to actin loading controls. C-PIK3CA-esiRNA treatment lead to reduced PIK3CA detection levels in all three xenograft tumor types (**Q-S**, third row from above) with even more pronounced suppression of PIK3CA protein by C-KRAS/PIK3CA-esiRNA combination in DLD1 and HT29 (**Q-R**, third row from above).

We next determined the knockdown efficiency of the esiRNA-targeted gene expression in tumors after the *in vivo* treatment. Therefore, we stained sections of paraffin embedded tumor samples for their KRAS and PIK3CA expression (Fig 4A–4P and Panels A-H in S1 Fig). KRAS expression was indeed decreased in C-KRAS-esiRNA treated DLD1 tumors (Fig 4C) or in combination C-KRAS/PIK3CA-esiRNA treatment (Fig 4G) compared to control tumors (Fig 4A). C-PIK3CA-esiRNA application did not decrease KRAS protein levels on DLD1 tumor sections (Fig 4E). These findings were consistent with the decreased KRAS protein expression detected in Western blot analysis using lysates of the same tumor treatment groups (Fig 4Q, upper panel and Fig A in S2 File). No differences concerning KRAS expression were visible in HT29 tumors on immunostained sections (Fig 4I, 4K, 4M and 4O), although we detected less KRAS expression in lysates of HT29 tumors treated with C-KRAS-esiRNA treatment (Fig 4R, upper panel and Fig B in S2 File).

Expression of PIK3CA was decreased in all treatment groups in DLD1 tumors including C-KRAS-esiRNA treatment (Fig 4D, 4F and 4H) compared to control-treated tumors (Fig 4B), which indicated a successful RNAi after C-PIK3CA-esiRNA application, but also hints at a presumed crosstalk between KRAS and PIK3CA expression. However, downregulation of PIK3CA upon C-KRAS-esiRNA treatment could not be observed in different lysates of these tumor treatment groups in Western blots, whereas PIK3CA expression was significantly downregulated in C-PIK3CA-esiRNA treated tumors (Fig 4Q, lower panel and Fig D-F in S2 File). The same effect was seen in stainings of HT29 tumors, in which C-PIK3CA-esiRNA and C-KRAS/PIK3CA-esiRNA treatment resulted in a lower expression level of PIK3CA (Fig 4N and 4P) compared to C-KRAS-esiRNA and control-treated tumors (Fig 4J and 4L), which is also reflected by the expression detected in the Western blot analysis (Fig 4R, lower panel). In lysates of SW480 tumors, we also identified a significant knockdown of KRAS in the C-KRAS-esiRNA group (Fig I in S1 File, upper panel) and of PIK3CA in the C-PIK3CA-esiRNA treatment group (Fig I in S1 File, lower panel). The Western blot results were reflected by the immunostainings in SW480 tumor sections (Panel A-H in S1 Fig). The treatment with C-KRAS-esiRNA and the C-KRAS/PIK3CA-esiRNA combination resulted in a decent reduction of cytoplasmic KRAS stain (Panel C and G in S1 Fig). Also, KRAS expression was diminished after C-PIK3CA-esiRNA treatment (Panel E in S1 Fig). PIK3CA expression decreased in SW480 tumors in all three treatment groups, again indicating a cross-talk between KRAS and PIK3CA signaling (Panels E, F, H in S1 Fig).

We conclude that the decreased tumor growth observed in the treatment groups is indeed based on the efficient downregulation of the intended and oncogenic factors KRAS and PIK3CA driven by an antibody-directed esiRNA delivery.

### Downstream effectors of KRAS and PIK3CA signaling are affected in xenograft tumors

**Fig 5:**
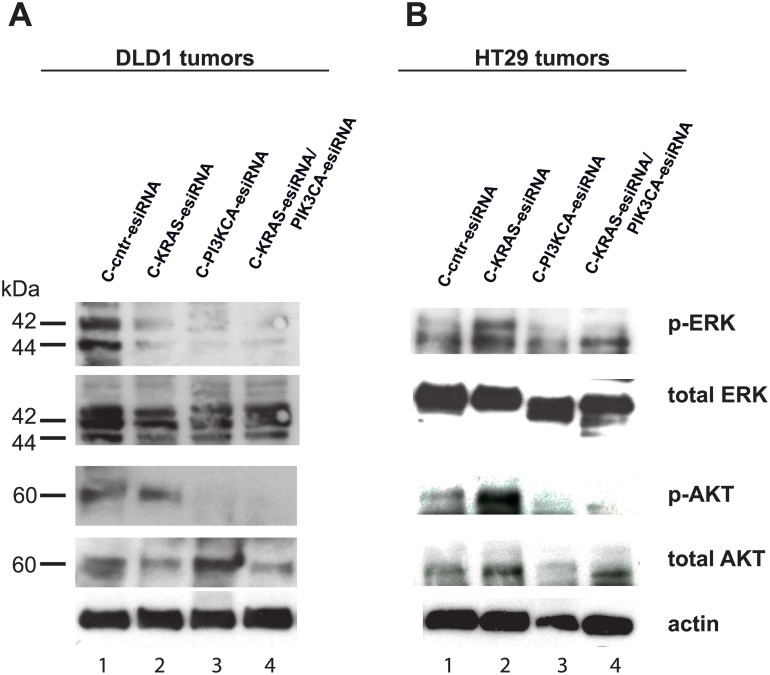
MAPK and AKT pathway inactivation by cetuximab-KRAS- and cetuximab-PIK3CA-esiRNA treatment. Tumor lysate samples from xenograft experiments were processed for SDS PAGE and probed in Western blots for expression of KRAS downstream MAPK pathway effector phospho-ERK and PIK3CA downstream phospho-AKT along with their unphosphorylated (total) counterparts in DLD1 (**A**) and HT29 (**B**) tumors. In DLD1 tumors (**A**) treated with C-KRAS-, C-PIK3CA- and C-KRAS/PIK3CA-esiRNA, there was a clear reduction of ERK phosphorylation visible in contrary to a control siRNA treatment (top row) and compared to total ERK levels, whereas BRAF-mutated HT29 tumors (**B**) treated with C-KRAS-esiRNA did not response in terms of ERK phosphorylation. Here, C-PIK3CA- and C-KRAS/PIK3CA-esiRNA treatment elicited reduced phosphorylation. The same western blot membranes were stripped and probed for phosphorylated AKT (p-AKT, **third row from above**) and total AKT (**fourth row from above**). Here, C-KRAS-esiRNA treatment did not change AKT phosphorylation in DLD1 (**A**) as well as HT29 (**B**) tumors as compared to control siRNA treatment, but C-PIK3CA-esiRNA treatment and C-KRAS/PIK3CA-esiRNA markedly reduced phosphorylation of AKT indicating deactivation of PI3K signaling pathway. Actin was detected as loading control for total protein.

Since KRAS and PIK3CA are mediators of EGFR signaling that affect downstream effectors via phosphorylation to deliver the signals, we analyzed the phosphorylation status of ERK (KRAS downstream factor) and AKT (PIK3CA downstream factor) in Western blot analysis in the tumors *ex vivo*. Here, as expected, ERK phosphorylation was downregulated upon KRAS inhibition in lysates of DLD1 xenograft tumors (Fig 5A, uppermost panel, lane 2 and 4), but not in lysates of KRAS-treated HT29 xenograft tumors (Fig 5B, uppermost panel, lane 4), while the total ERK expression is largely unchanged (Fig 5A and 5B). Interestingly, inhibition of PIK3CA expression also lowered the phosphorylation of ERK (Fig 5A and 5B, lane 3), indicating again a certain crosstalk between these signaling pathways. At the end of this signaling cascade, the expression of c-MYC can be effected, as addressed below.

As anticipated the phosphorylation of AKT decreased upon PIK3CA-RNAi in lysates of DLD1 and HT29 xenograft tumors (Fig 5A and 5B, lower panels, lane 3 and 4). Total AKT was detected in these samples (Fig 5A and 5B).

The transcription factor c-MYC is a known downstream effector of ERK signaling [38], which is regulated on the transcriptional level and has a long standing history [39–41] as one of the most potent oncogenic factors in cancer. In the previous study [35], we identified c-MYC as a target for cetuximab mediated KRAS knockdown.

**Fig 6:**
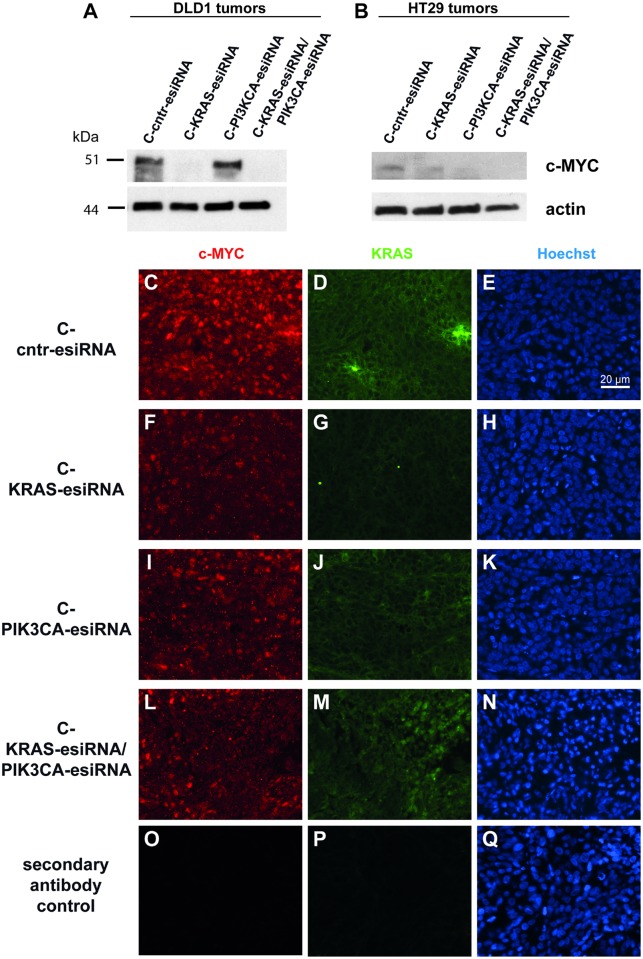
MAPK pathway suppression by cetuximab-KRAS siRNA treatment regulates c-MYC transcription factor levels in treated tumor samples. **(A-B)** Cetuximab-siRNA treated tumor lysate samples from xenograft experiments were processed for Western blot and probed for c-MYC protein expression controlled by actin loading control. In DLD1 tumor samples (**A**) treated with C-KRAS-esiRNA and C-KRAS/PIK3CA-esiRNA, c-MYC protein level almost completely disappeared (top row), whereas the c-MYC protein expression in HT29 tumors was lower and was not consistently reduced by C-KRAS-, but by C-PIK3CA-esiRNA and C-KRAS/PIK3CA-esiRNA treatment. **(C-Q)** DLD1 tumor samples showing a clear c-MYC dependency on active MAPK-signaling as shown above in Western blot were paraffin embedded, sectioned and processed for double-immunofluorescence imaging. Simultaneous detection of c-MYC (red) and KRAS (green) protein on the same sections and frames with respective primary antibodies showed a correlation between markedly reduced KRAS cytoplasmic stain by C-KRAS-esiRNA treatment (**G**) and diminished c-MYC nuclear stain (**F**). C-PIK3CA-esiRNA treatment did not reduce KRAS (**J**) or c-MYC (**I**) levels. The combination treatment C-KRAS/PIK3CA-esiRNA showed less pronounced effects as compared to C-KRAS-esiRNA on both KRAS (**M)** and c-MYC expression (**L**). The right column shows Hoechst nuclear stain of the left and middle frames.**O-Q** show secondary antibody controls.

Here, we tested c-MYC levels in lysates of C-control, C-KRAS-esiRNA, C-PIK3CA-esiRNA, and C-KRAS/PIK3CA-esiRNA treated DLD1 and HT29 tumors from our xenografts. Western blots exhibited major downregulation of c-MYC protein upon C-KRAS-esiRNA and C-KRAS/PIK3CA-esiRNA treatment, but not C-PIK3CA-esiRNA treatment in DLD1 tumors (Fig 6A), whereas HT29 tumor reacted to treatment in a different manner. At first, c-MYC was expressed to a lesser extent in HT29, then c-MYC was not consistently downregulated by C-KRAS-esiRNA treatment, but rather by C-PIK3CA-esiRNA, and C-KRAS/PIK3CA-esiRNA treatment, indicating the involvement of the PI3K pathway here (Fig 5B). We postulated that the drastic c-MYC downregulation in DLD1 must be visible also in immunohistochemical stainings of the tumors and proved this as shown in Fig 6C–6N. Paraffin embedded DLD1 tumors were sectioned, rehydrated, blocked and subjected to c-MYC and KRAS immunofluorescence accompanied by Hoechst nuclear stain to visualize tissue integrity. Indeed, there was a decent reduction in c-MYC nuclear stain in case of C-KRAS-esiRNA treatment of DLD1 tumors (Fig 6F), meanwhile KRAS levels were also reduced by KRAS knockdown (Fig 6G). In C-PIK3CA-esiRNA treated tumors, no reduction of c-MYC and KRAS was detected (Fig 6I and 6J). The treatment with C-KRAS/PIK3CA-esiRNA resulted in an intermediate reduction of both KRAS and c-MYC, accompanied with the restriction that these C-KRAS/PIK3CA-esiRNA treated tumors were extremely small and prone to necrotic effects (Fig 6L and 6M and seen by condensed or even denucleated Hoechst nuclear stain in central area of N).

Taken together, results hint to an influence of c-MYC in our modelled MAPK pathway abrogation by C-KRAS-esiRNA treatment in DLD1 and in the PI3K pathway interference by C-PIK3CA-esiRNA treatment in HT29. Further studies are needed to elucidate a possible function of c-MYC.

## Discussion

A recent key goal in cancer therapy is “precision cancer medicine” [28,29], which complements “individualized” or “personalized” therapy. The genomic landscape within a cancer is examined in particular concentrating on "driver" events, with the hope to delineate a tailored-made targeted therapy. With the insights of molecular tumor-biology, more specific agents than chemotherapeutic cytotoxic drugs have indeed been developed, and targeted therapy concepts have been combined with conventional therapeutics. The introduction of small molecule inhibitors against specific oncogenic factors such as imatinib in BCR-ABL positive chronic lymphocytic leukemia paved the road towards successful targeted cancer therapy. However, the application of one single agent against one oncogenic product will most likely not be effective in many solid tumors with a high heterogeneity and abundance of different mutations [29,42]. The EGF receptor and signaling pathways downstream of the EGF receptor were identified as major drivers of metastatic CRC growth, and a subset of CRC patients benefit from the inhibition of EGFR by monoclonal antibodies such as cetuximab [3]. However, mutations in the KRAS, NRAS, BRAF and/or PIK3CA genes downstream EGFR can prevent a successful cetuximab therapy, and even cetuximab-sensitive tumors frequently relapse via different mechanisms of acquired drug resistance.

RNA interference might serve as an opportunity to address some of these problems. siRNA can be designed against the expression of virtually every gene, even against a specific point mutation within an oncogenic factor or the breakpoint of an oncogenic fusion such as EWS-FLI1 in Ewing sarcoma [34]. Formerly “undruggable” targets like KRAS can be downregulated very efficiently by esiRNA [35]. Endoribonuclease prepared esiRNAs show very low off-target effects, compared to classic siRNAs [43,44]. The conceptual problems of this therapy form appeared in form of low stability of siRNA due to enzymatic cleavage and renal clearance before getting to the place of action. We propose a concept of application where these disadvantages can be overcome by complexing esiRNA to cancer-cell specific antibodies [35]. In this report, we have combined the cetuximab-coupled esiRNA against KRAS with esiRNA targeting PIK3CA. In our preclinical xenograft model, the combination of both esiRNAs is more effective than the single esiRNA application to inhibit tumor growth caused by simultaneous interference with KRAS and PIK3CA expression. Moreover, we identified the expected downstream targets of KRAS and PIK3CA signaling as downregulated, thus indicating a functional signaling pathway silencing. We conclude that RNAi by the systemic application of antibody-complexed esiRNA is efficient *in vivo*. This experimental treatment option is a promising new approach to react in a precise and individual manner to a patient’s requirements according to composition of (i) tumor oncogene and (ii) surface markers by a targeted therapy in a modular fashion.

This therapeutic option might be accompanied by much less adverse events and side effects than the application of small molecule inhibitors. Indeed, the cetuximab dosage we applied here (4 mg cetuximab/kg body weight) was considerably lower than in other studies [45,46]. Of note, cetuximab-protamine/siRNA complex offers no elevated toxicities over protamine-conjugated cetuximab in various CRC cell lines, as shown previously [35] and shows the same, mutation-dependent cetuximab resistance effects as known from various literature sources [47–49]. This gives a hint that the efficacy and safety of the carrier antibody are not changed by the protamine/siRNA conjugation method.

For our experimental regimen, we chose wild type KRAS and PIK3CA as esiRNA targets, although the definition of point-mutation specific RNAi should also be addressed in a refinement study. Small molecules against KRAS—wild type or mutated—are not easily available to date, RNAi approaches have been proposed but lacked cell determining components so far [50]. We and others applied stabilized anti-KRAS esiRNA *in vivo* and saw a strong decrease in tumor growth [35,50], which might be an excellent option to overcome the undruggable state of mutated active KRAS [32,51]. The multiple attempts to generate and safely apply an effective PIK3CA inhibitor did not lead to an FDA-approved therapy in solid tumors yet and the development of a specific RNAi-based therapy is worthwhile. The downregulation on mRNA level has the chance to lead to an irreversible apoptotic and/or anti-proliferative signal that kills the tumor cell before it acquires resistant properties. Small molecular drugs developed against PIK3CA usually compete for ATP binding, which is a reversible process and consequently at equilibrium with the non-inhibited form [12,22,28]. The development of resistance mechanisms against small molecular drugs by mutation of active sites are known, whereas resistance mechanisms against RNAi have not yet been elucidated in detail.

In our experimental setup, PIK3CA inhibition by RNAi led to decreased tumor growth independent of the PIK3CA mutational status of the cell line: both PIK3CA wild type and mutant cell lines reacted upon treatment with PIK3CA esiRNA. Partially, this is also true for mutational status of KRAS. In detail, HT29 are not KRAS- but BRAF-mutated (V600E) leading to deregulated BRAF activation. As BRAF is downstream of KRAS interference with KRAS expression should exhibit little effect, which was the case in the C-KRAS-esiRNA treated HT29 xenograft. Here, a combination of BRAF interfering siRNAs and PIK3CA should exhibit maximum effect, a concept that will be targeted in future projects. Similar additive effects were shown by others applying nanoparticle-packed siRNA-treatments against PIK3CA and KRAS [50]. Its seems that intensive crosstalk and by-pass reaction between the RAS-RAF-ERK-MAPK pathway and the PI3K-AKT-mTor pathway requires an inhibition of both arms to be really efficient, which was seen in different approaches in CRC [52] and also in non-small cell lung cancer cell (NSCLC) xenograft tumors treated with small molecules [53].

We were intrigued by the rigorous c-MYC protein reduction after cetuximab-mediated KRAS knockdown in the treated tumors, which parallels our results in the treated cell lines in the previous project [35]. c-MYC expression is known to have a strong interdependence with ERK activation and as an integrator or sensor of ERK and PIK3CA signals [54]. In detail, activated RAS signaling through effector ERK resulted in phosphorylation and stabilization of c-MYC by attenuation of its ubiquitin-mediated protein degradation mechanism in melanoma [55]. The responsible phosphorylation sites in c-MYC have been elucidated through mutagenesis in fibroblasts [38] and detected as phosphorylated in mammary tumors [41]. Conversely, inactivation of ERK cascade by small molecular drugs abrogated c-MYC expression in rhabdomyosarcoma [56]. We see that c-MYC expression is reduced by MAPK pathway inactivation (Fig 6), and to a minor degree also in the c-PIK3CA treatment of HT29 tumors. Taken together, results hint to an important involvement of c-MYC in our modelled MAPK pathway abrogation by cetuximab-KRAS esiRNA treatment in DLD1 and in the PI3K pathway interference by cetuximab-PIK3CA esiRNA treatment in HT29.

In conclusion, further analysis and development of antibody-stabilized and -transported esiRNA against driver oncogenes is a very promising approach to overcome different problems in cancer therapy and should be studied within a wider scope.

## Supporting information

S1 Fig Treatment of CRC xenografts with cetuximab-protamine-siRNA markedly reduces siRNA target gene expression. **A-H**. Paraffin sections from SW480 tumors were prepared for immunohistochemical (IHC) analysis for siRNA targets KRAS and PIK3CA with antibody detecting KRAS (A, C, E, G) and PIK3CA (B, D, F, H) combined with suitable secondary antibodies and stained with diaminobenzidine (DAB) and hematoxylin and pictures taken at 20x magnification from regions without signs of necrosis. The application of KRAS siRNA coupled to cetuximab-sulfo-SMCC-protamine (C-KRAS-esiRNA) markedly reduced KRAS immunostaining **(C)** in SW480 tumors compared to control C-cntr-esiRNA **(A)**. The treatment with C-PIK3CA-esiRNA reduced PIK3CA immunostaining in SW480 (**F**) compared to control C-cntr-esiRNA in both cell lines (**B**). Interestingly, C-KRAS-esiRNA treatment also reduced PIK3CA staining in SW480 tumors (**D**). The combination treatment of tumors with KRAS- and PIK3CA-esiRNAs (C-KRAS/PIK3CA-esiRNA) resulted in reduced KRAS and PIK3CA staining (**G-H**). The C-PIK3CA-esiRNA monotherapy (**E-F**) lead to diminished PIK3CA staining as well as KRAS staining. **I**. Western blots indicating siRNA target gene induced protein synthesis control in xenograft tumor tissue of cetuximab-protamine-esiRNA treated mice. Tumor tissue was processed for western blot as described, applied to SDS-PAGE, blotted and exposed for immunodetection by antibodies raised against KRAS, PIK3CA and actin as loading control. Application of cetuximab-protamine coupled to KRAS-esiRNA (C-KRAS-esiRNA) reduced KRAS protein levels in SW480 (**upper row**) tumor xenografts as compared to controls (actin row). In addition, C-KRAS-esiRNA treatment showed indifferent PIK3CA expression effect in SW480 (**lower row**) as compared to actin loading controls. C-PIK3CA-esiRNA treatment lead to reduced PIK3CA detection levels (**I**, third row from top).
(TIF)

S2 Fig Densitometry analysis to quantify the Western blot bands, which are shown in Figs 4 and 5 for representative examples. Western blots were scanned and analysed with ImageJ. Pixel density was normalized to control esiRNA. Error bars and significance indexes show a statistical analysis of biological replicates of n>2. **A, B, C** show KRAS knockdown response: KRAS intensity / actin intensity, normalized to knockdown response in c-cntr-esiRNA. **D, E, F** show PIK3CA knockdown response: PIK3CA intensity / actin intensity, normalized to knockdown response in c-cntr-esiRNA. **G, H, I** show ERK phosphorylation status in comparison to total ERK: phosphor-ERK intensity / total-ERK intensity, normalized to knockdown response in c-cntr-esiRNA. **J, K** show AKT phosphorylation status in comparison to total AKT: phosphor-AKT intensity / total-AKT intensity, normalized to knockdown response in c-cntr-esiRNA. * = significance p < 0.05; ** p < 0.01; *** p<0.001.
(EPS)

S1 Table Mutation status of the CRC cell lines. (DOCX)
