# Supplementary figures and images for: Downregulation of PIK3CA via antibody-esiRNA-complexes suppresses human xenograft tumor growth
Source: PLoS One. 2018 Jul 12;13(7):e0200163. doi: 10.1371/journal.pone.0200163 (PMC6042707; doi:10.1371/journal.pone.0200163)

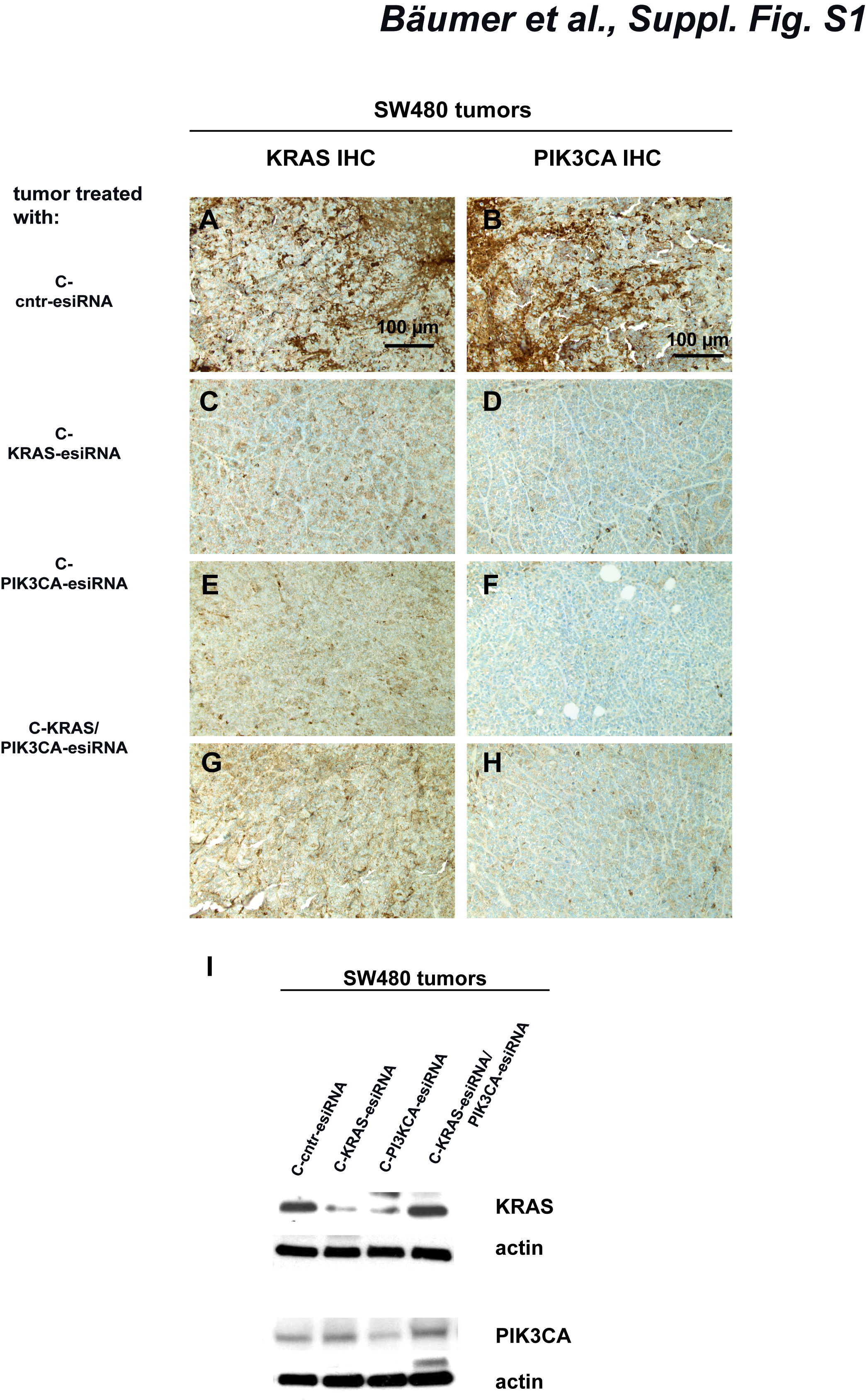

Supplement: S1 Fig — A-H. Paraffin sections from SW480 tumors were prepared for immunohistochemical (IHC) analysis for siRNA targets KRAS and PIK3CA with antibody detecting KRAS (A, C, E, G) and PIK3CA (B, D, F, H) combined with suitable secondary antibodies and stained with diaminobenzidine (DAB) and hematoxylin and pictures taken at 20x magnification from regions without signs of necrosis. The application of KRAS siRNA coupled to cetuximab-sulfo-SMCC-protamine (C-KRAS-esiRNA) markedly reduced KRAS immunostaining (C) in SW480 tumors compared to control C-cntr-esiRNA (A). The treatment with C-PIK3CA-esiRNA reduced PIK3CA immunostaining in SW480 (F) compared to control C-cntr-esiRNA in both cell lines (B). Interestingly, C-KRAS-esiRNA treatment also reduced PIK3CA staining in SW480 tumors (D). The combination treatment of tumors with KRAS- and PIK3CA-esiRNAs (C-KRAS/PIK3CA-esiRNA) resulted in reduced KRAS and PIK3CA staining (G-H). The C-PIK3CA-esiRNA monotherapy (E-F) lead to diminished PIK3CA staining as well as KRAS staining. I. Western blots indicating siRNA target gene induced protein synthesis control in xenograft tumor tissue of cetuximab-protamine-esiRNA treated mice. Tumor tissue was processed for western blot as described, applied to SDS-PAGE, blotted and exposed for immunodetection by antibodies raised against KRAS, PIK3CA and actin as loading control. Application of cetuximab-protamine coupled to KRAS-esiRNA (C-KRAS-esiRNA) reduced KRAS protein levels in SW480 (upper row) tumor xenografts as compared to controls (actin row). In addition, C-KRAS-esiRNA treatment showed indifferent PIK3CA expression effect in SW480 (lower row) as compared to actin loading controls. C-PIK3CA-esiRNA treatment lead to reduced PIK3CA detection levels (I, third row from top). (TIF) [file pone.0200163.s001.tif]
