# Supplementary material for: Downregulation of PIK3CA via antibody-esiRNA-complexes suppresses human xenograft tumor growth
Source: PLoS One. 2018 Jul 12;13(7):e0200163. doi: 10.1371/journal.pone.0200163 (PMC6042707; doi:10.1371/journal.pone.0200163)
Supplement: S1 Table — (DOCX) [file pone.0200163.s003.docx]

**S1 Table**

| **Cell line** | **siRNA vs. mutation/ activated gene-product** | **Signal transduction pathway** | **Downstream marker** | **Reference** |
| --- | --- | --- | --- | --- |
| HT-29 | BRAF V600E | RAS/RAF/MEK/ERK | p-ERK, c-myc | 34, 35 |
|  | PIK3CA P449T | PI3-Kinase | p-AKT | 34, 35 |
| SW480 | KRAS G12V | RAS/RAF/MEK/ERK | p-ERK, c-myc | 34 |
| DLD1 | KRAS G13D | RAS/RAF/MEK/ERK | p-ERK, c-myc | 12 |
|  | PIK3CA 545K;D549N | PI3-Kinase | p-AKT | 34 |
